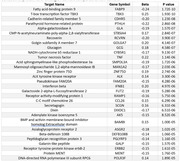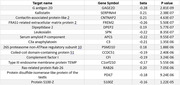# Circulating proteins associated with rate of cognitive decline based on APOE genotype

**DOI:** 10.1002/alz.086739

**Published:** 2025-01-09

**Authors:** Alison E Fohner, Jennifer A. Brody, Colleen Sitlani, Diane Xue, W.T. Longstreth, Michelle Odden, Thomas R. Austin, Robert Edgardo Gerszten, Emily H. Trittschuh, Bruce M. Psaty, Sudha Seshadri

**Affiliations:** ^1^ University of Washington, Seattle, WA USA; ^2^ Stanford University, Stanford, CA USA; ^3^ Beth Israel Deaconess Medical Center, Boston, MA USA; ^4^ University of Washington, School of Medicine, Seattle, WA USA; ^5^ Geriatric Research, Education, and Clinical Center, Veterans Affairs Puget Sound Health Care System, Seattle, WA USA; ^6^ Glenn Biggs Institute for Alzheimer’s & Neurodegenerative Diseases, University of Texas Health Sciences Center at San Antonio, San Antonio, TX USA

## Abstract

**Background:**

APOE genotype is the most important genetic risk factor for Alzheimer’s disease (AD), but whether it affects the proteins associated with AD risk is unclear. Circulating proteins may reveal biology underlying pathologic processes.

**Methods:**

We evaluated log2 standardized levels of 4979 proteins quantified using modified aptamer technology [SomaScan] in plasma from 2725 participants in the Cardiovascular Health Study who were free of dementia and stroke. Participants were recruited from 4 sites in the United States and plasma samples were obtained in 1992/1993. Cognitive decline was measured with annual modified mini mental state exams (3MSE) and digit symbol substitution tests (DSST) over 7 years. APOE genotype was classified as e4 carrier, e2 carrier, or non‐carrier. Adjusting for sex, age, race, education, and study site, we used linear mixed effects modeling to evaluate whether the association of each protein with rate of cognitive decline differed based on APOE genotype. We ran two models: one comparing APOE e4 to the other genotypes and another comparing APOE e2 to the other genotypes. A p‐value of 2.1x10^‐5^ was considered significant.

**Results:**

Mean age of participants was 74 years, 61% were female, 15% were Black, 25% were APOE e4 carriers, 17% were APOE e2 carriers, and mean cognitive follow‐up was 5.2 years. For 3MSE, 38 proteins had significant interactions with time and APOE e4 carrier status (Table 1) and 15 proteins with APOE e2 carrier status (Table 2). For DSST, only 3 proteins had significant interaction with time and APOE e4 carrier status and none with APOE e2. Proteins included those involved in lipid transport, neuronal differentiation, immune response, and cell signaling.

**Conclusions:**

Large‐scale proteomic profiling identified circulating proteins differentially associated with cognitive decline based on APOE genotype. Many of the proteins are involved in pathways previously implicated in AD risk. Replication and other characterization are needed to clarify how APOE genotype may affect mechanisms involved in cognitive decline and AD risk.